# Clinical features and treatment outcome of elderly multiple myeloma patients with impaired renal function

**DOI:** 10.1002/jcla.22888

**Published:** 2019-04-19

**Authors:** Jin Chen, Hui Liu, Lijuan Li, Zhaoyun Liu, Jia Song, Guojin Wang, Huaquan Wang, Erbao Ruan, Kai Ding, Zonghong Shao, Rong Fu

**Affiliations:** ^1^ Department of Hematology Tianjin Medical University General Hospital Tianjin China

**Keywords:** age, bortezomib, multiple myeloma, renal impairment, survival

## Abstract

**Background:**

Renal impairment (RI) is a most common complication of multiple myeloma (MM), which is associated with an increased risk of early death and worse survival.

**Methods:**

We retrospectively analyzed clinical features and outcomes of 77 MM patients over 70 years old and compared the differences between with and without RI groups.

**Results:**

The percentage of elder MM patients with RI was 61%. Hemoglobin level was a protective factor (OR = 0.954, *P* = 0.033), while creatinine and hypertension were hazards (OR = 1.288, *P* < 0.001 and OR = 30.12, *P* = 0.008). And the percentages of patients with mild‐to‐moderate RI and moderate‐to‐severe RI were 40.4% and 59.6%. Complete remission (CR) rate was higher in patients treated with bortezomib (33.3%) than those with non‐bortezomib treatment (3.33%) (*P* = 0.007). Meanwhile, CRrenal was higher in patients with bortezomib (58.3%) than non‐bortezomib treatment (22.2%) (*P = *0.025). The median OS of the patients with RI treated with bortezomib was longer than those with non‐bortezomib regimens (15.0 vs 6.0 months, *P* = 0.001). The same result was observed in the patients with moderate‐to‐severe RI (13.0 vs 6.0 months, *P* = 0.007). The median OS of the patients with RI receiving the bortezomib regimens (15 months) was longer than those with non‐bortezomib regimens (6.0 months) (*P* = 0.001).

**Conclusion:**

Hemoglobin is a protective factor in elder patients with RI, while creatinine and hypertension were hazards. The median OS of elderly patients with RI was worse, and bortezomib can improve the CR rate in these patients.

## 1 INTRODUCTION

Multiple myeloma (MM) is a common hematologic malignancy, which is characterized by malignant expansion of monoclonal plasma cells in the bone marrow. The clinical features in the majority of MM include calcemia, renal failure, anemia, and bone disease (CRAB). MM is a kind of disease of the elderly with a median age of presentation in the early 70s.^1^, ^2^ Although World Health Organization (WHO) defines “elderly” as older than the age of 65 years, the aging is a evidently heterogeneous phenomenon.^3^ The incidence of MM increases steadily with advanced age.^4^ Meanwhile, the incidence of various complications in elderly MM patients also is increased. As we all know, renal impairment (RI) is a most common complication of MM that can be present at diagnosis or emerge during therapy.^5^, ^6^ RI can be elicited by various factors, such as infections, non‐steroidal anti‐inflammatory drugs, nephrotoxic antibiotics, iodinated contrast media, hypercalcemia, tumor lysis syndrome, myeloma cell infiltration of the kidney, and renal vein or artery thrombosis, and, frequently, by clonotypic light chains.^7^ The presence of RI places the patients at higher risk for complications after anti‐myeloma treatment and is associated with an increased risk of early death.^8^ The MM patients with advanced age are usually excluded from clinical trials due to poor performance status (PS), various complications, and socioeconomic reasons.^9^ Therefore, limited scientific data are available regarding the clinical characteristics and treatment outcomes in this group of patients.^10^, ^11^ To clarify these issues, we compared the clinical characteristics and treatment outcomes of all patients admitted and treated at our hospital over 70 years old MM patients in recent years.

## 2 PATIENTS AND METHODS

A total of 77 consecutive patients with newly diagnosed MM from October 2010 to December 2015 were retrospectively analyzed. Twenty patients refused chemotherapy and only received supportive treatment. Treatment responses were evaluated according to the international uniform response criteria for multiple myeloma.^12^ The reduction or suspension of treatment was determined according to the decision of the physician, the patient, or their family. Multiple baseline characteristics of the patients were collected from the medical records (Tables 1 and 2). Response to treatment was assessed by the International Myeloma Working Group (IMWG) criteria.^12^, ^13^

**Table 1:** The clinical and laboratory characteristics between the patients without RI and with RI (n = 77)

Clinical features	Pts without RI	Pts with RI	*P*‐Value
n (%)	30 (39.0)	47 (61.0)	
Median age (y)	73.5(70‐86)	75 (70‐87)	0.468
Male, n (%)	16 (53.3)	34 (44.2)	0.141
Diabetes, n (%)	3 (10.0)	9 (19.1)	0.348
Hypertension, n (%)	11 (36.7)	30 (63.8)	0.034
Light chain MM, n (%)	4 (13.3)	11 (23.4)	0.277
IgG κ, n (%)	5 (16.7)	9 (19.1)	0.783
IgG λ, n (%)	3 (10.0)	11 (23.4)	0.137
IgG, n (%)	3 (10.0)	5 (10.6)	0.137
IgA κ, n (%)	7 (23.3)	5 (10.6)	0.134
IgA λ, n (%)	3 (10.0)	3 (6.4)	0.564
IgA, n (%)	1 (3.3)	0 (0)	0.208
Non‐secreting type	4 (13.3)	3 (6.4)	0.301
ISS stage, n (%)			
Ⅰ	5 (16.7)	1 (2.1)	0.000***
Ⅱ	13 (43.3)	6 (12.8)	0.000***
Ⅲ	9 (30.0)	39 (50.6)	0.000***
Leukocyte (×10^9^/L)	6.39 ± 3.85	5.36 ± 2.85	0.114
Hemoglobin (g/L)	95.43 ± 25.15	77.21 ± 23.23	0.007**
Thrombocyte (×109/L)	161.00 ± 90.48	137.64 ± 65.76	0.227
Albumin (g/L)	31.13 ± 6.45	33.13 ± 6.21	0.180
Globulin (g/L)	52.29 ± 23.07	51.43 ± 26.37	0.883
Alkaline phosphatase (U/L)	75.07 ± 34.46	71.91 ± 32.39	0.690
BUN (mmol//L)	6.27 ± 1.61	12.77 ± 6.98	0.000***
Creatinine (μmol/L)	68.59 ± 16.43	276.43 ± 201.27	0.000***
GFR (mL/min)	139.02 ± 60.99	44.56 ± 28.96	0.000***
LDH (U/L)	182.17 ± 70.94	228.96 ± 118.21	0.036*
Calcium (mmol/L)	2.33 ± 0.51	2.46 ± 0.34	0.173
β2‐MG (mg/L)	4.73 ± 4.46	12.35 ± 7.14	0.000***
Myeloma cell (%)	33.54 ± 20.26	37.15 ± 22.61	0.499
FISH (n = 17)	n = 5	n = 12	0.942
p53, n (%)	1 (20)	1 (8.3)	
RB1, n (%)	1 (20)	2 (16.7)	
del13, n (%)	2 (40)	5 (41.7)	
1q‐, n (%)	4 (80)	6 (50.0)	
Abnormal karyotype (n = 35)	2/11	2/24	0.575

BUN, blood urea nitrogen; FISH, fluorescence in situ hybridization; GFR, glomerular filtration rate; LDH, lactic dehydrogenase; MM, multiple myeloma; n, number; Pts, patients; RI, renal impairment; β2‐MG, β2‐microglobulin.

* *P* < 0.05.

** *P* < 0.01.

*** *P* < 0.001.

**Table 2:** The clinical and laboratory characteristics between the patients with mild‐to‐moderate RI and moderate‐to‐severe RI (n = 47, GFR < 90 mL/min)

Clinical features	Pts with GFR ≥ 60 mL/min	Pts with GFR < 60 mL/min	*P*‐Value
n (%)	19 (40.4)	28 (59.6)	
Median age (y)	74 (70‐87)	75 (70‐83)	0.341
Male, n (%)	14 (73.7)	20 (71.4)	0.865
Diabetes, n (%)	2 (10.5)	20 (71.4)	0.000***
Hypertension, n (%)	10 (52.6)	20 (71.4)	0.188
Light chain MM, n (%)	3 (15.8)	8 (44.4)	0.310
IgG κ, n (%)	4 (21.1)	5 (17.9)	0.785
IgG λ, n (%)	2 (10.5)	9 (32.1)	0.086
IgG, n (%)	3 (15.8)	2 (25)	0.345
IgA κ, n (%)	4 (21.1)	1 (3.6)	0.056
IgA λ, n (%)	1 (5.3)	2 (25)	0.796
IgA, n (%)	0 (0)	0 (0)	‐‐
Non‐secreting type, n (%)	2 (10.5)	1 (3.6)	0.338
ISS stage, n (%)			
Ⅰ	1 (5.3)	0 (0)	0.079
Ⅱ	5 (26.3)	2 (3.6)	0.079
Ⅲ	13 (68.4)	26 (92.9)	0.079
Leukocyte (×10^9^/L)	5.24 ± 3.95	5.45 ± 1.86	0.833
Hemoglobin (g/L)	83.53 ± 26.42	72.93 ± 20.17	0.126
Thrombocyte (×10^9^/L)	131.58 ± 71.87	141.75 ± 62.30	0.608
Albumin (g/L)	34.84 ± 6.76	31.96 ± 5.63	0.120
Globulin (g/L)	50.53 ± 26.51	52.04 ± 26.75	0.850
ALP (U/L)	65.05 ± 24.26	76.74 ± 36.73	0.232
BUN (mmol//L)	8.16 ± 1.51	15.90 ± 7.51	0.000***
Creatinine (μmol/L)	106.63 ± 10.66	391.64 ± 186.77	0.000***
GFR (mL/min)	76.71 ± 6.45	22.74 ± 13.41	0.000***
LDH (U/L)	211.95 ± 88.75	240.93 ± 135.55	0.419
Calcium (mmol/L)	2.41 ± 0.16	2.50 ± 0.41	0.367
β2‐MG (mg/L)	6.33 ± 3.10	16.60 ± 6.02	0.000***
Myeloma cell (%)	32.54 ± 22.03	40.51 ± 22.87	0.247
FISH (n = 13)	n = 4	n = 9	0.877
p53, n (%)	0 (0)	1 (11.1)	
RB1, n (%)	1 (25)	2 (22.2）	
del13, n (%)	1 (25)	4 (44.4）	
1q‐, n (%)	2 (50)	4 (44.4）	
Abnormal karyotype (n = 22)	0/9	2/13	0.217

ALP, alkaline phosphatase; BUN, blood urea nitrogen; FISH, fluorescence in situ hybridization; GFR, glomerular filtration rate; LDH, lactic dehydrogenase; MM, multiple myeloma; n, number; Pts, patients; RI, renal impairment; β2‐MG, β2‐microglobulin.

* *P* < 0.05.

** *P* < 0.01.

*** *P* < 0.001.

Renal function and renal response to therapy were assessed. Renal function was assessed by estimated glomerular filtration rate (eGFR), calculated using Chronic Kidney Disease Epidemiology Collaboration (CKD‐EPI) creatinine equation.^14^ The degree of renal impairment (RI), based on values of eGFR measured as mL/min/1.73 m^2^, was graded as follows: G1 (normal renal function), ≥90; G2 (mild RI), 60‐89; G3a (mild‐to‐moderate RI), 45‐59; G3b (moderate‐to‐severe RI), 30‐44; G4 (severe RI), 15‐29; and G5 (renal failure), <15 or on dialysis.^14^ In our study, the group of no RI was rated when eGFR≥90, the group of mild‐to‐moderate RI was rated when eGFR≥60, and the group of moderate‐to‐severe RI group was rated when eGFR<60. Renal response was defined as complete (CRrenal), partial (PRrenal), minor (MRrenal), or no (NRrenal), according to the criteria formulated by the IMWG. In particular, CRrenal was defined as an increase in eGFR from <50 to 60 mL/min/1.73 m^2^ or better, PRrenal an increase from <15 to 30‐59 mL/min/1.73 m^2^, and MRrenal an increase from <15 to 15‐29 mL/min/1.73 m^2,^ or from 15‐29 to 30‐59 mL/min/1.73 m^2^ 15; NRrenal was defined as GFR did not increase to achieve the above criteria.

In all patients, 57 patients received chemotherapy, including non‐bortezomib and bortezomib regimens. In non‐bortezomib group, 30 patients received TD, MP, or MTD regimen. In bortezomib group, 27 patients received VD or VCD ±T regimen. Meanwhile, supportive therapies were given in these patients, including blood transfusion, anti‐infection, and hemodialysis, if necessary. All patients were evaluated for at least two cycles of treatment, and we evaluated the treatment response after two cycles.

Progression‐free survival (PFS) was defined as the time from the start of any kind of treatment to the date on which progression from best response or death occurred, whichever came first. Overall survival (OS) was calculated from the time of first diagnosis of symptomatic myeloma to the time of death. The end of the follow‐up is death, loss, or December 2015.

Clinical baseline characteristics of two groups were described as mean ± SD for normal distributed continuous variables and median for non‐normally distributed variables. Categorical variables were described as percentages. The distribution of continuous variables in two groups was compared by independent‐samples *t* test for normal distributed variables, by non‐parametric test for non‐normal distributed variables, and by analysis of variance for categorical variables. Kaplan‐Meier survival curves were constructed, and difference of survival rates was tested by log‐rank test. Statistical significance was defined as a *P*‐value of <0.05 for all tests. All statistical analyses were performed by SPSS 21.0.

## 3 RESULTS

### 3.1 Baseline characteristics

Table 1 shows the clinical and laboratory characteristics between the patients with or without RI. The percentages of patients without RI and with RI were 39% and 61%, respectively. There were significant differences in hypertension, ISS stage, hemoglobin, BUN, creatinine, GFR, LDH, and β2‐microglobulin between the two groups, while no differences in age, sex, diabetes, light chain isotype, IgA isotype, leukocyte, thrombocyte, globulin, albumin, alkaline phosphatase, calcium, or myeloma cell. Hemoglobin levels were protective factors (OR = 0.954, *P* = 0.033), while creatinine and hypertension were hazards (OR = 1.288, *P* < 0.001 and OR = 30.12, *P* = 0.008). Table 2 shows the clinical and laboratory characteristics between the patients with mild‐to‐moderate RI and moderate‐to‐severe RI, which were 40.4% and 59.6%, respectively. There were significant differences in diabetes, BUN, creatinine, GFR, and β2‐microglobulin between the two groups.

### 3.2 Treatment outcome

Fifty‐seven patients received chemotherapy, including TD, VAD, and MP (non‐bortezomib group, n = 30) or VD, VCD, and VTD (bortezomib group, n = 27, 2 patients had no therapeutic evaluation). Table 3 shows the treatment outcomes of the two groups. CR rate was higher in bortezomib group (non‐bortezomib and bortezomib; 3.33% and 33.3%, respectively, *P* = 0.007). Furthermore, there were 21 patients whose GFR is <50mL/min received chemotherapy. We found that CRrenal was higher in bortezomib group (non‐bortezomib and bortezomib; 22.2% and 58.3%, respectively, *P = *0.025) (Table 4).

**Table 3:** Treatment response of all the patients received bortezomib and non‐bortezomib (n = 55)

Treatment response	Non‐bortezomib n (%)	Bortezomib n (%)
CR	1 (3.33)	9 (33.3)
PR	20 (66.7)	15 (55.6)
SD	4 (13.3)	0 (0)
PD	5 (16.7)	1 (3.7)

CR, complete remission; n, number; PD, progressive disease; PR, part remission; SD, stable disease.

**Table 4:** Treatment response of patients with RI received bortezomib and non‐bortezomib (n = 21)

Treatment response	Non‐bortezomib n (%)	Bortezomib n (%)
CRrenal	2 (22.2)	7 (58.3)
PRrenal	1 (11.1)	1 (8.3)
MRrenal	1 (11.1)	4 (33.3)
NRrenal	5 (55.5)	0 (‐)

CRrenal, complete renal response; MRrenal, minor renal response; n, number; NRrenal, no renal response; PRrenal, partial renal response.

### 3.3 Progression‐free and overall survival

**Figure 1:**
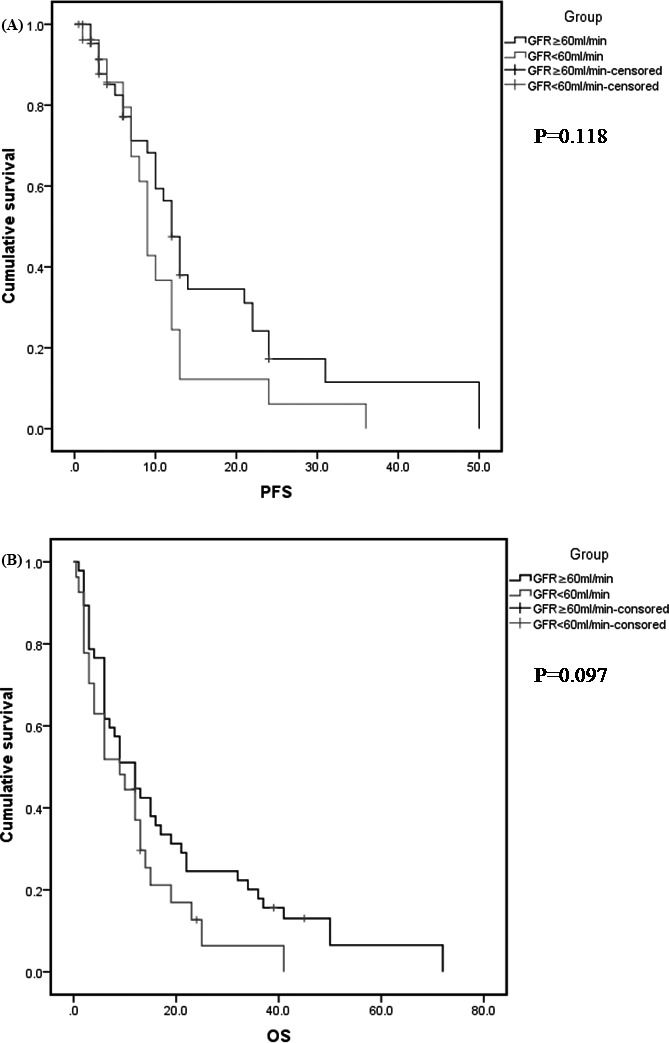
Survival of patients according to the different GFR groups. A, Progression‐free survival; (B) overall survival

**Figure 2:**
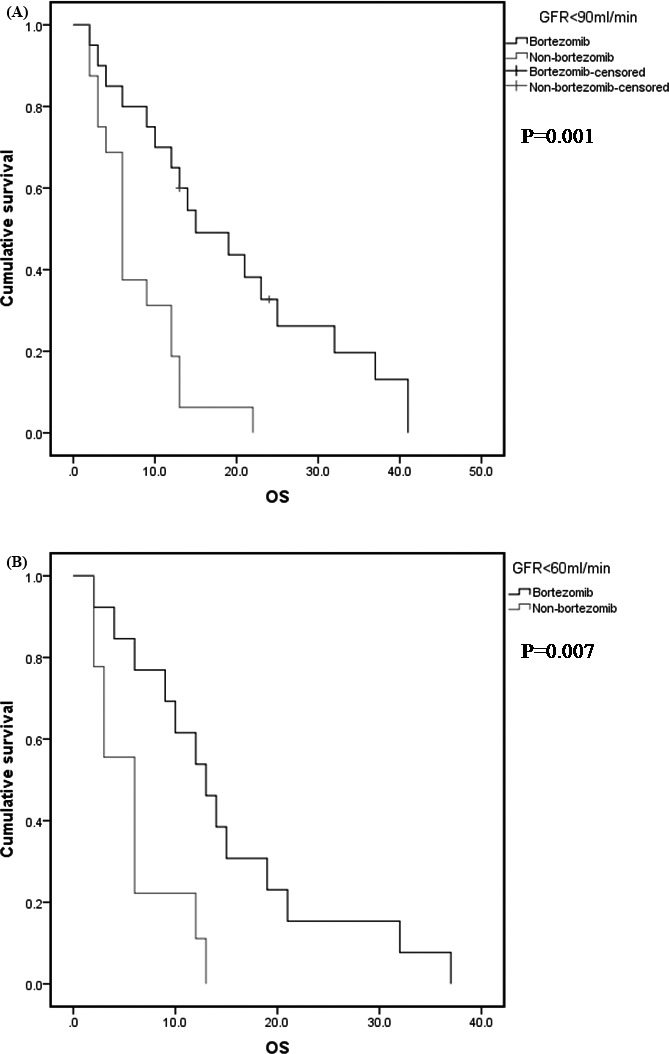
Overall survival of patients according to the different GFR groups treating with the bortezomib. A, Overall survival of the group with RI treating with the bortezomib‐containing regimens and non–bortezomib‐containing regimens. B, Overall survival of the group with moderate‐to‐severe RI treating with the bortezomib‐containing regimens and non–bortezomib‐containing regimens

At a median follow‐up of 12 months (range: 2‐72 months), median duration of progression‐free survival (PFS) and overall survival (OS) for all patients was 10 months and 9 months. To explore the impact of renal function on survival, PFS and OS were calculated and compared according to mild RI and severe RI (Figure 1). The median PFS of the group with mild‐to‐moderate RI and moderate‐to‐severe RI was 10.0 and 9.0 months, and the median OS of these two groups was 12.0 and 9.0 months. Log‐rank analysis indicated that there was no significant difference in PFS and OS between these two groups, while we found PFS and OS of the group with mild‐to‐moderate RI were longer than those with moderate‐to‐severe RI. Furthermore, the median OS of the group with RI and with moderate‐to‐severe RI treating with the bortezomib‐containing regimens was longer than those with non–bortezomib‐containing regimens, 15.0 vs 6.0 months and 13.0 vs 6.0 months, respectively, *P* = 0.001 and *P* = 0.007) (Figure 2).

## 4 DISCUSSION

During the past decade, although drugs for MM have undergone significant improvement, MM is still difficult to be cured. RI is a common complication in patients with MM, and its incidence increases in patients with relapsed or refractory MM.^16^ MM is a disease of the elderly, and most patients with newly diagnosed MM are more than 65 years old. An analysis in 3107 newly diagnosed MM patients demonstrated that RI was a direct or major influencing factor in at least one‐third of early deaths in these patients.^8^ Thus, despite the use of novel drugs, high early mortality in MM patients with RI remains significant, indicating that rapid and effective intervention is needed.

There are many complications in the elderly patients with MM, such as hypertension, coronary heart disease, and diabetes. Among 77 MM patients enrolled in our study, we found that 63.8% of MM patients with RI have hypertension, which is significantly higher than those without RI. After logistic regression modeling analysis, we found that hypertension was a hazard in elderly MM patients.

Regarding the optimal therapy for patients with RI, currently available data indicate that bortezomib is probably the preferred drug.^17^, ^18^ For the first time, achievement of CR was not necessarily followed by a prolonged survival, if the treatment was stopped in relation to drug toxicity profile.^19^ Bortezomib has excellent activity in MM at any stage of the disease and is synergistic with other drugs, which led to several combination strategies. VMP was proven superior to MP in response rate, CR rate, median TTP (time to progression), and OS, even over all cytogenetic and renal failure subgroups.^20^ The improvement in RI to a near‐normal range (CrCL ≥60 mL/min) observed in the majority of MM patients in the current study suggests that bortezomib may be a particularly useful therapy in this setting. In our study, CR rate was higher in patients treated with bortezomib. As the similar result, CRrenal was higher in bortezomib group. Among the patients ≥70 years old, patients without RI showed significantly longer PFS and OS compared to those with RI. The OS of patients during the treatment of bortezomib‐containing regimens in mild‐to‐moderate RI and moderate‐to‐severe RI group was longer than those without RI.

In conclusion, we showed that elderly MM patients with RI, using first line of bortezomib‐containing regimens treatment, can achieve higher CR and CRrenal rates, and prolong their survival.
